# Contributions of Ultrastructural Studies to the Knowledge of Filamentous Fungi Biology and Fungi-Plant Interactions

**DOI:** 10.3389/ffunb.2021.805739

**Published:** 2022-01-24

**Authors:** Franco Faoro, Antonella Faccio, Raffaella Balestrini

**Affiliations:** ^1^Dipartimento di Scienze Agrarie e Ambientali, Università di Milano, Milan, Italy; ^2^Consiglio Nazionale delle Ricerche, Istituto per la Protezione Sostenibile delle Piante, Turin, Italy

**Keywords:** ultrastructure (electron microscopy), fungi, plant-fungus interactions, plant pathogens, mycorrhizal fungi

## Abstract

Since the first experiments in 1950s, transmission electron microscopy (TEM) observations of filamentous fungi have contributed extensively to understand their structure and to reveal the mechanisms of apical growth. Additionally, also in combination with the use of affinity techniques (such as the gold complexes), several aspects of plant-fungal interactions were elucidated. Nowadays, after the huge of information obtained from -omics techniques, TEM studies and ultrastructural observations offer the possibility to support these data, considering that the full comprehension of the mechanisms at the basis of fungal morphogenesis and the interaction with other organisms is closely related to a detailed knowledge of the structural features. Here, the contribution of these approaches on fungal biology is illustrated, focusing both on hyphae cell ultrastructure and infection structures of pathogenic and mycorrhizal fungi. Moreover, a concise appendix of methods conventionally used for the study of fungal ultrastructure is provided.

## Introduction

Transmission electron microscopy (TEM) observations of filamentous fungi have been determinant not only for understanding their structure but, even more important, for revealing the mechanisms of apical growth. The first recorded work on the ultrastructure of fungal cells dates back to 1958 and concerned *Trametes versicolor* (L.:Fr.) Pilàt (formerly *Polystictus versicolor*), a wood decay basidiomycete (Girbardt, 1958). However, most of the initial data on ascomycetes and basidiomycetes ultrastructure derive mainly from the studies of Moore and McAlear in 1961–1963, which described the plasma membrane, nuclei, mitochondria, Golgi apparatus the type of septa in these two main groups of filamentous fungi [i.e., *Neobulgaria pura* (Pers.: Fr.) Petr. belonging to the Ascomycota and *Uromyces caladii* Sch. belonging to the Basidiomycota]. At that time, the most used fixative was potassium permanganate (KMnO_4_), which could easily permeate into fungal walls but did not result in adequate preservation of cell ultrastructure. Later on, fixation with osmium tetroxide, in addition to KMnO_4_, allowed to better define other organelles, such as ribosomes, endoplasmic reticulum and mitochondrial cristae (Ceruti et al., 1964). However, it was the introduction of double fixation with glutaraldehyde and osmium tetroxide (OsO_4_) that resulted in a much clearer definition of hyphae ultrastructure, i.e., showing various type of apical and subapical vesicles and their connection with the cytoskeleton in different fungal species, such as the Ascomycota *Ascodesmis nigricans* Tiegh. and the Basidiomycota *Armillaria mellea* (Vahl.: Fries) (Grove and Bracker, 1970). Ten years later, a newer technique, termed freeze substitution, dramatically improved the knowledge of fungal ultrastructure (i.e., of the Ascomycota *Fusarium acuminatum* Ellis and Everh), further clarifying the mechanisms of apical growth (Howard and Aist, 1979). By this technique, living fungal structures are cold fixed at −190°C into liquid propane and then transferred to a mixture of cold (−80°C) acetone, osmium tetroxide and uranyl acetate (C_4_H_6_O_6_U), before being raised back to room temperature and cryo-sectioned or embedded in conventional plastic resins. Another significant advance in knowledge was the development in the 1980s of immunocytochemical techniques for the detection of macromolecules by TEM (Bendayan, 1984). By this technique it was possible to have a deeper insight not only in fungal wall structure but also in the mechanisms of apical growth, particularly regarding the trafficking of the different macromolecules involved in hyphae elongation and in the role of the Spitzenkorper (Roberson et al., 2010).

Nowadays knowledge on ultrastructure of fungi achieved by all the above-mentioned techniques and their contributions to improve knowledge in fungal biology are illustrated herewith together with the advancements in sample preparation and TEM observations. Description mainly focuses to the two phyla of Ascomycota and Basidiomycota, the most studied fungi and, besides hyphae cell ultrastructure, infection structures of pathogenic and mycorrhizal fungi are included. Finally, in the Supplementary File, a concise list of fixations and embedding protocols useful to study fungal cells by TEM is reported.

## Hyphae Apical and Subapical Compartments

**Figure 1:**
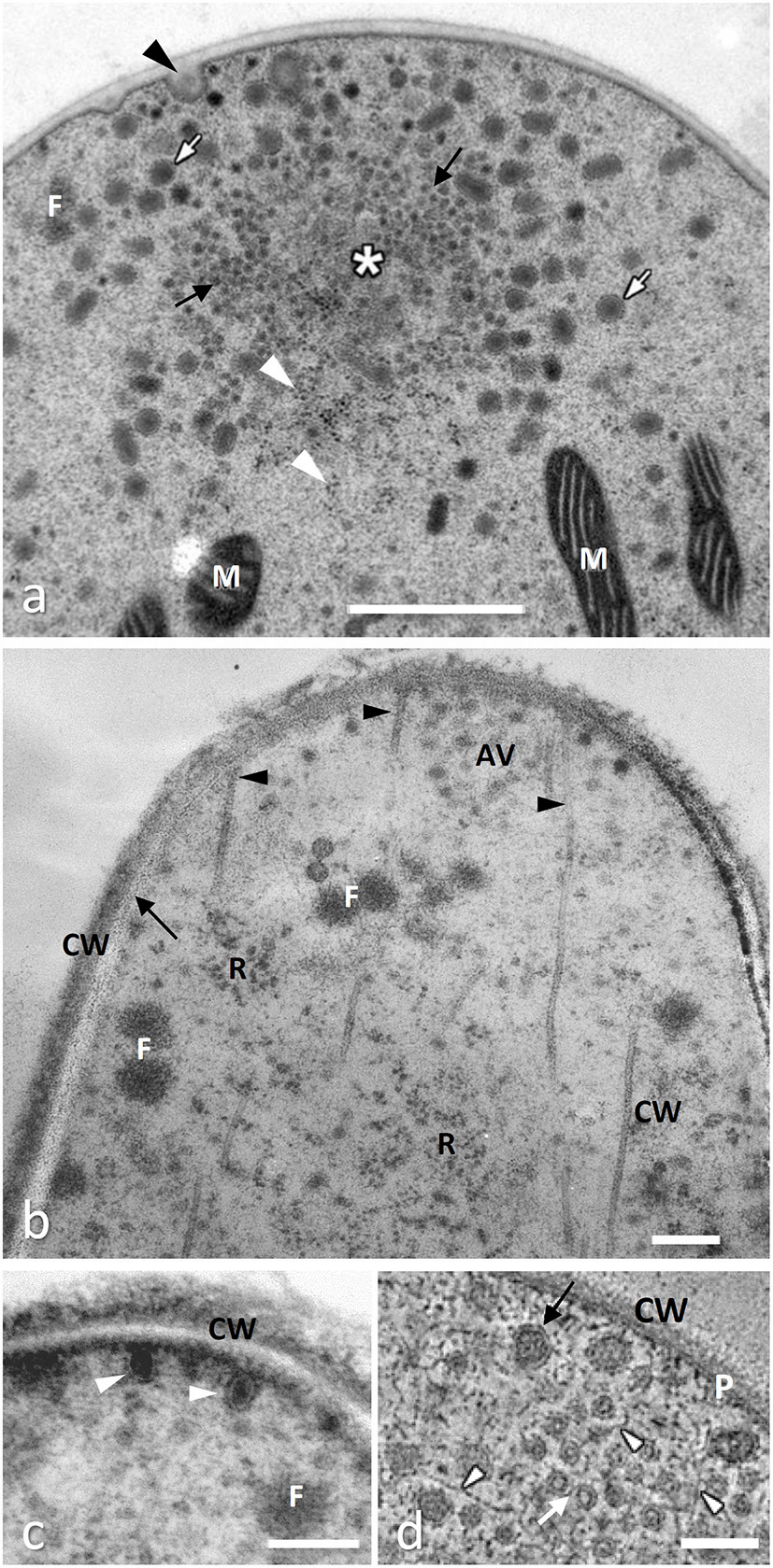
Extreme hyphal tips cryofixed and freeze substituted. **(a)** *Neurospora crassa* (Ascomycota) showing a typical Spitzenkörper formed by a cluster of apical vesicles around a core mainly composed of actin filaments (asterisk); actin filaments are also present in smaller clusters (filasomes, F) usually close to cell membrane; vesicles are of different size and electron density, the smallest (microvesicles, black arrows) are apparently surrounded by larger ones (macrovesicles, white arrows) and many ribosomes (white arrowheads); vesicles exocytosis is often visible (black arrowhead) while mitochondria (M) are rare in the extreme tip (from Roberson, 2020, with permission of the publisher). **(b)** *Tuber melanosporum*, Ascomycota, showing apical vesicles (AV) organized in a possibly simplified Spitzenkörper and microtubules (arrowheads) running to the plasma membrane which appears linear (arrow) between the cell wall (CW) and the cytoplasm, rich in ribosomes (R); (from Amicucci et al., 2011, with permission of the publisher). **(c)** An enlarged serial section of the same hyphal tip as in **(b)** showing chitosome-like vesicles (arrowheads) delivering they cargo of chitin synthase to the plasma membrane to build cell wall (CW) which appears not fully organized yet. **(d)** *Aspergillus nidulans* G.Wint, Ascomycota: TEM tomography of the hyphal tip: by this technique microvesicles (white arrows) and apical vesicles (black arrow) appear clearly embedded in a matrix of actin microfilaments (white arrowheads); the double layer of the plasma membrane (P) is also clearly visible; (from Hohmann-Marriott et al., 2006, with permission of the publisher). All bars, 0.2 μm.

**Figure 2:**
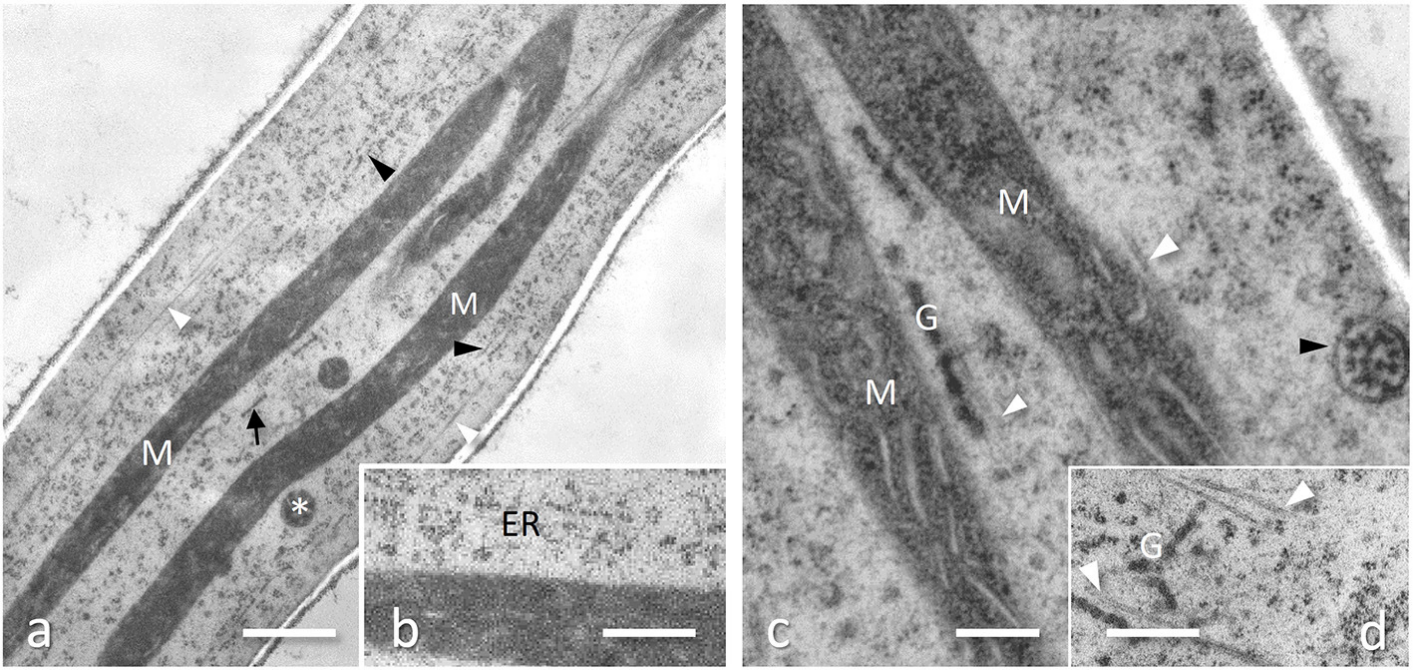
Subapical region of *Tuber melanosporum* hyphae, Ascomycota, cryofixed and freeze substituted. **(a)** Numerous very elongated mitochondria (M) running along to the longitudinal axis of the hyphae, together with a well-developed ER (black arrowheads), numerous microtubules (white arrowheads) and sausage-like strings of Golgi cisternae (arrow). Bar, 1.0 μm. **(b)** Enlargement of the ER in Figure 5. Bar, 0.5 μm. **(c)** Mitochondria (M) showing flat cristae, in close association with microtubules (white arrowhead) and Golgi (G), whose secretory vesicles may be enclosed in membrane-bound multi-vesicular bodies (black arrowhead) for a faster delivery to hyphae tip; from Amicucci et al., with permission of the publisher. Bar, 0.2 μm. **(d)** Ramified cisternae string of Golgi (G) among microtubules (white arrowheads). Bar, 0.5 μm.

The first images of ultrathin sections of hyphae tips in filamentous fungi, obtained by chemical fixation, demonstrated that this cell area is characterized by the presence of numerous vesicles, often bound to cell membranes and by the absence of other organelles. These vesicles were observed in all the studied fungal species of different phyla, such the Ascomycota *Ascodesmis nigricans* Tiegh., *Neurospora crassa* Shear and Dodge, *Aspergillus niger* Tiegh. and *Fusarium oxysporum* Schlectendahl as well as in *Armillaria mellea* Vahl.: Fries belonging to Basidiomycota, and *Gilbertella persicaria* (E.D. Eddy) Hesselt belonging to Mucoromycotina. Interestingly, they were also present in the hyphae tips of fungal-like organisms, i.e., the Oomycota *Pythium aphanidermatum* (Edson) Fitzp. and *Globisporangium ultimum* (Trow) Uzuhashi, Tojo and Kakish (formerly *P. ultimum*, Edson) (Grove and Bracker, 1970; Grove et al., 1970). The huge number of vesicles was soon associated to the need of material for the rapid growth of the tip, for the extension of cell membrane and wall. Afterwards, cryofixation and freeze substitution resulted in much more details of hyphae tip and subapical compartments (Hoch, 1986; Welter et al., 1988). By this technique it was possible to differentiate the numerous vesicles either by their size, content, location, and possible origin, thus substantially contributing to understand the mechanisms underlaying both hyphae behavior and apical growth (Knauf and Mendgen, 1988). The cluster of vesicles located very close to the hyphae tip (AVC, apical vesicles cluster) is known since a long time as Spitzenkörper because when observing actively growing hyphae at phase-contrast light microscope it appears as a dark body, clearly regulating direction and branching of hyphae but also their growth, as it disappears when the latter is stopped (Brunswick, 1924). The Spitzenkörper is then the coordinator of polarized hyphal extension (Bartnicki-Garcia, 1990) and its organization is now well-documented by TEM (Roberson et al., 2010). It consists of a group of vesicles of different size and electron opacity encircling a vesicle-free area (Figure 1a). Although the content of these vesicles is difficult to define, due to technical difficulties associated with their differential isolation, some cytochemical studies demonstrated that a subset of vesicles 40–70 nm in diameter contain chitin synthase, therefore named chitosomes (Figures 1b,c) (Sietsma et al., 1996) and some others β-1,3-glucan synthase (Sanchez-Leon and Riquelme, 2015). Other vesicles very likely contain plasma membrane and cell wall components, proteins involved in membrane docking and fusion and signaling molecules (Virag and Harris, 2006) while some specific vesicles, called filasomes, contain actin filaments (Figures 1a,b) (Howard, 1981). At high magnification, and especially in cryofixed specimens, microtubules, 25 nm in diameter, are seen running parallel to the hyphae axis and through the apical vesicles up to the plasma membrane (Figure 1b). Together with microfilaments they contribute to the fast delivery of vesicles originated from the Golgi tubules located further behind in the cytoplasm. The involvement of actin microfilaments both in movement and delivery of vesicles has been clearly demonstrated by tomographic studies at TEM of ultrathin sections of hyphae apex (Figure 1d; Roberson et al., 2010). Finally, many ribosomes are present in/around the Spitzenkörper (Figures 1a,b) indicating that intensive protein synthesis occurs at the hyphal tip (Grove and Bracker, 1970). The subapical region close to the tips is characterized by numerous mitochondria (Figure 2a) that are involved into the generation of a gradient across cell membrane for driving absorption of nutrients at hyphal tip (Roberson et al., 2010). Intermingled with mitochondria there are a well-developed ER, peculiar Golgi apparatus and longitudinally oriented microtubules, often running close to mitochondria, as detailed in the next section. A clear ultrastructural equivalent of the Spitzenkörper as a dense, spheroidal cluster of vesicles, and cytoskeletal components has been found at the tips of growing hyphae in members of the Ascomycota and Basidiomycota (Roberson et al., 2010), while it is not common in other phyla, although it has been described in the phylum Blastocladiomycota, and specifically in *Allomyces macrogynus* (Vargas et al., 1993). More recently, a protocol of cryofixation by plunging mycelium samples into liquid nitrogen-cooled propane (Hoch, 1986; Howard and O'Donnell, 1987), and subsequently processing them for freeze substitution and embedding as described in McDaniel and Roberson (2000), has been applied to highlight the ultrastructure of *Tuber melanosporum* Vittad., detecting the characteristic subcellular components of the hyphal tip in septate filamentous fungi (Amicucci et al., 2011). Interestingly, despite the presence in *T. melanosporum* of almost all the genes involved in filamentous hyphal growth, TEM observations allowed to show that a clear equivalent of the Spitzenkörper, was not identified in its hyphal tips, suggesting that the slower hyphal extension rates that are characteristic of this fungal species require a small number of secretory vesicles, not necessarily well-organized in a fully developed Spitzenkörper as those of other faster growing species (Amicucci et al., 2011).

## Fungal Plasma Membrane and Cell Organelles

**Figure 3:**
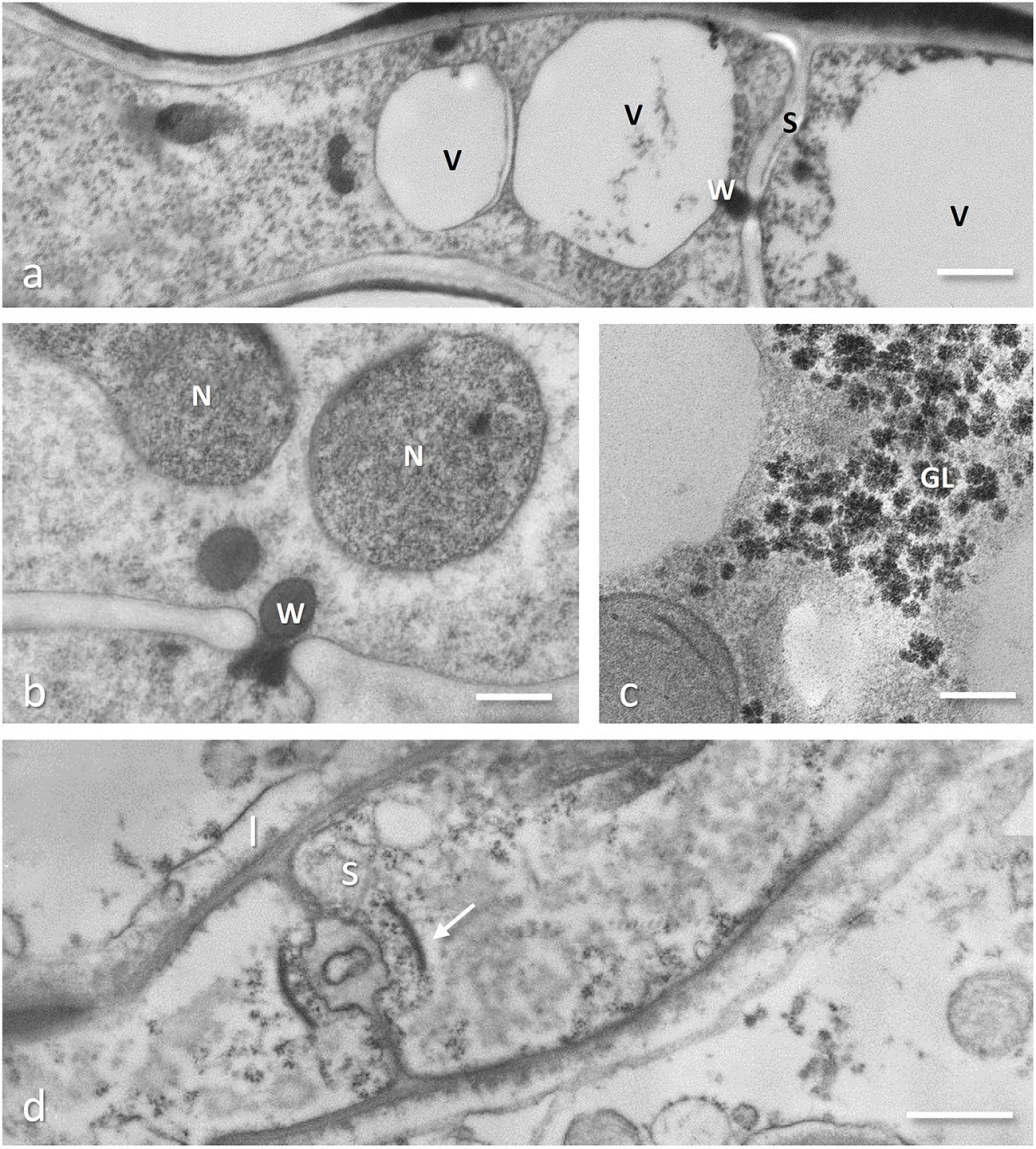
Cryofixed and freeze substituted hyphae (*Tuber melanosporum*, **a,b**; *Gigaspora margarita*, **c**) and conventionally chemical fixed (*Rhizoctonia*-like mycorrhizal fungus, **d**). **(a)** An old part of the hyphae showing two cells, separated by a septum (S) with the central pore plugged by a Woronin body; the cell on the left has two possibly melting vacuoles (V) while the one on the right show a single large vacuole occupying the whole cell lumen. Bar = 0.5 μm. **(b)** The septum pore plugged with a Woronin body, besides avoiding loss of protoplasm through the damaged part of the hyphae, can also impair migration of nuclei (N) between adjacent cells. Bar = 0.2 μm. **(c)** Glycogen (GL) deposits in an intracellular hypha of the arbuscular mycorrhizal fungus *Gigaspora margarita*, showing the typical aggregation of the single granules (preparation described in Balestrini et al., 1996a). Bar = 0.2 μm. **(d)** Rhizoctonia-like mycorrhizal orchid fungus showing a dolipore septum (S) typical of Basidiomycota surrounded by a flattened and imperforated parenthosome (arrow) characteristic of these orchid mycorrhizal species; the interfacial compartment bounded by the plant plasma membrane is also visible (I). Bar = 1.5 μm.

It is probably not necessary to underline the importance of transmission electron microscopy for the relevant contribution to highlight fungal ultrastructure. True fungal plasma membrane, as in all eukaryotes, is composed by a lipid bilayer and associated proteins and sterols, thus in thin sections at TEM is indistinguishable from plant and animal membranes. However, in contrast with the latter which contain phytosterols and cholesterol, the sterol of fungal membranes is ergosterol (Rodrigues, 2018). In chemically fixed fungal cells, plasma membrane appears slightly wavy; however, this could be an artifact as in cryofixed and freeze-substituted samples it is perfectly flat (Figures 1b,d). The nucleus is generally small (see Figure 3b, next section), around 1–2 μm in diameter, surrounded by a double membrane that remains intact through almost all steps of mitosis, possibly to avoid diffusion of nuclear content into the protoplasm that often contain several nuclei. Like all eukaryotic cells, the secretory pathway includes the endoplasmic reticulum (ER) and the Golgi apparatus. From an ultrastructural point of view ER membranes appear generally aligned along the hyphal longitudinal axis (Figures 2a,b) and are particularly present in the subapical region of the hyphae, where the protein synthesis is very intense both to produce structural proteins but also enzymes for hyphae wall building and for the degradation of substrates in the surrounding environment. These proteins and enzymes are modified through the Golgi cisternae, particularly regarding their glycosylation pattern, then delivered to the site of wall expansion packed into vesicles. The Golgi apparatus in fungi is quite peculiar and very different from the stack of flat cisternae as in plants and animals. Indeed, it is formed by sausage-like strings (Figures 2a,c), sometimes ramified (Figure 2d) and often located close to longitudinally running microtubules, possibly for a fast delivery of vesicles to the hyphal apex (Figures 2c,d). Because of this peculiar form, at TEM it is difficult to localize Trans-Golgi Network (TGN). However, confocal laser scanning microscopy with fluorescent probes clearly demonstrated the importance of this compartment in recycling chitin synthase. In fact, this fundamental enzyme after exocytosis at hyphae tip is re-uptake in the subapical region via endocytic internalization and sent to TGN where it accumulates before being re-delivered to the apex (Hernandez-Gonzalez et al., 2018). To facilitate their delivery to hyphal tip, the smallest vesicles, such as chitosomes, may also be clustered in larger multivesicolar bodies (Figure 2c) (Roberson and Fuller, 1988; Sánchez-León et al., 2011). Mitochondria are characterized by flat cisternae as in plants and animals (Figure 2c), but different from the tubular ones that are characteristic of fungus-like organisms of the phylum Oomycota (Powell and Blackwell, 1995). In the subapical region they are very elongated (Figure 2a) possibly because of the rapid hyphae growth. Vacuoles are present as numerous rounded structures in hyphae region further back from the apex. Though at TEM they appear as static structure (Figure 3a), they are indeed very dynamic as they merge and regenerate continuously as demonstrated by light microscopy of living hyphae after fluorescent staining (Rees et al., 1994). They can even become narrow tubules that can travel along hyphae through septa up to the tip thus forming a sort of channel that allows metabolite movement backwards and forwards (Rees et al., 1994). Besides storage and recycling of cell metabolites, fungal vacuoles are supposed to facilitate hyphae elongation pushing forward the protoplasm to the tip. In the older hyphae regions vacuoles generally merged in a larger one occupying almost the whole cell volume (Figure 3a). Ascomycota also have a peculiar peroxisome-derived organelle, often observed close to septa, and termed Woronin body (Figure 3b), from the Russian botanist that discovered it. This is a dense core microbody surrounded by a single membrane that can plug septal pores to avoid loss of protoplasm through the damaged part of the hyphae (Figure 3b). Finally, another typical inclusion observed in the cytoplasm of filamentous fungi is formed by glycogen aggregates being this multibranched polysaccharide a form of energy storage as in animals (Figure 3c). It is worth noting the attempt of Kuga et al. (2008) to use TEM in association with multiple methods to describe the ultrastructure and the polyphosphate distribution in rapidly frozen and freeze-substituted germ tubes of the arbuscular mycorrhizal (AM) fungus *Gigaspora margarita* Gerd. & *Trappe*, AM fungi belong to the Glomeromycotina subphylum (Joseph et al., 2016), and in AM symbiosis the supply of phosphorus from the fungi is one of the most important benefits to the host plant. These authors showed that vacuoles are predominant organelles occupying most of the cell volume and therefore have the potential for phosphorus storage.

## Fungal Cell Wall

**Figure 4:**
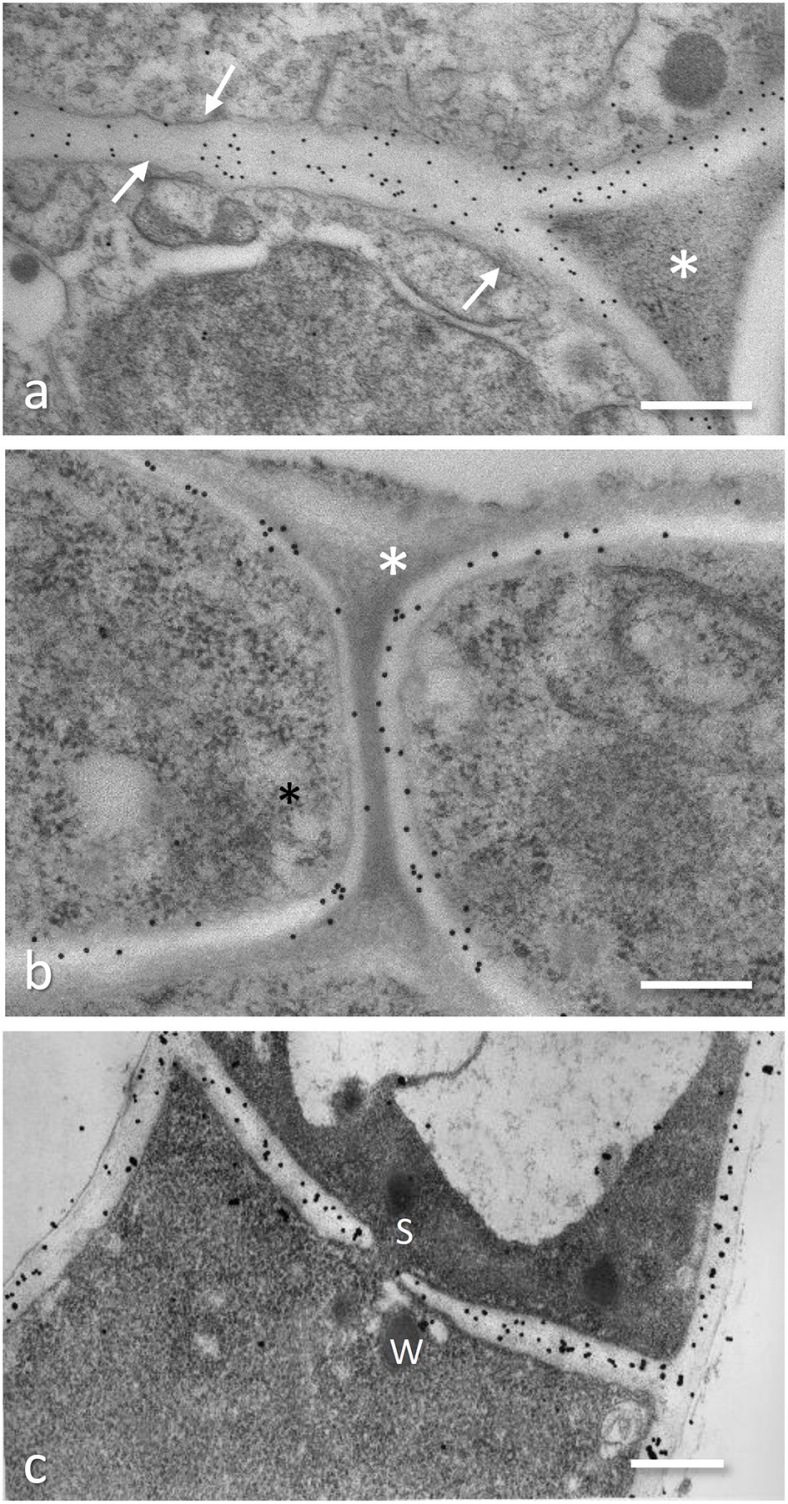
Immunogold localization of fungal cell wall components on ultrathin sections of specimen fixed and embedded following the protocol 2 in the Appendix. **(a,b)** Localization of β-1,3-glucans on *Tuber melanosporum* fruiting body hyphae and on the interface *Tuber melanosporum*-*Corylus avellana* ectomycorrhizae: 20 nm gold granules are present on the fungal cell wall (arrows). In ectomycorrhizae **(b)**, the fungal cell wall is labeled in both the mantle hyphae (D) and in the hyphae progressing between the root cells during the Hartig net establishment (E). No gold granules are present on the electron-dense material with a triangle shape that fills the space among the hyphae (asterisk). Bar, 0.3 μm. **(c)** Localization of chitosan on cell wall and septum (S) of *Blumeria graminis* f.sp. *hordei* by a chitosanase-20 nm gold probe; courtesy of D. Maffi. Bar, 0.5 μm.

The fungal cell wall is a dynamic structure that protects fungal cells from changes in osmotic pressure and other environmental stresses, while allowing to interact with the environment and other organisms (Garcia-Rubio et al., 2020). Besides to maintaining cell shape and integrity during environmental stress, the cell wall plays a role as a signaling center, activating signal transduction pathways within fungal cells, mediating interactions with the external environment through receptors that trigger a complex cascade of signals inside the cell (Bowman and Free, 2006). Because of its essential biological role, unique composition and structural organization, and the absence in mammalian cells of its main components, the cell wall is a suitable target for the development of novel antifungal agents (Latgé, 2007, 2010; Lima et al., 2019). Although its composition changes depending on the taxonomic group, this structure is usually composed of chitin (i.e., a structural polymer of β-1,4-N-acetylglucosamine), glucans (mainly branched β-1,3-glucans), and proteins that are extensively cross-linked together to form a complex network representing the structural basis of the cell wall (Bowman and Free, 2006; Gow et al., 2017). Particularly, glucans are the most abundant polysaccharides in the fungal cell walls. They are either lineal or branched, amorphous or microfibrillar, and their structures are variable, with the glucose moieties that may be joined through alpha (α) or beta (β) linkages (Ruiz-Herrera and Ortiz-Castellanos, 2019). Melanin can be also present in some species and structures (Free, 2013; Garcia-Rubio et al., 2020). Since the first studies, TEM ultrastructural observations allowed to obtain information on the architectural complexity of fungal cell walls. After the report of a fibrillar texture of insoluble components in fungal cell walls of *Phycomyces* spp. sporangiophores by Frey-Wyssling and Mühlethaler (1950) and Aronson and Preston (1960) observed that a stratification exists in cell walls with the fibrillar skeletal components that are oriented toward the inner surface, while the outer surface is smooth and composed of amorphous material (Figures 1c,d). Generally, the innermost layer consists of a core of covalently attached branched β-1,3-glucan and chitin, and the outer layers are more heterogeneous, and its composition can vary among species and morphotypes (Gow et al., 2017; Garcia-Rubio et al., 2020). The cell wall of the fungal pathogen *Candida albicans sensu lato* is formed by an amorphous inner skeletal layer of β-1,3- and β-1,6-glucan, and chitin, and an outer fibrillar layer mainly containing highly mannosylated cell wall proteins. The architecture of these two layers can be visualized at the electron microscopy level, but the observed structure of the wall has not been defined precisely in chemical terms yet. Transmission electron microscopy and tomography could provide the precise structure, location and molecular sizes of the cell wall components and predictions of the cell wall models using mutants and agents that perturb the normal cell wall structure could confirm the observations (Roberson et al., 2010). However, as above reported, fungal cell wall is a dynamic structure, subjected to constant modifications, depending on the culture conditions and developmental stage, i.e., during cell expansion and division in yeasts, and during spore germination, branching and septum formation in filamentous fungi as well as in response to environmental stresses (Gow et al., 2017). TEM observations were widely used to study morphological features of fungal cell wall in diverse stages during growth. It has been also observed that structure, composition, and properties of the cell wall vary along the length of a polarized hypha (Riquelme et al., 2018). At the growing hyphal tip, the cell wall is thin (~50 nm) and plastic (Figures 1–4), though it becomes thicker (< ~250 nm) and more rigid with further cell wall synthesis and crosslinking of its components. Septa are also composed of typically thick cell walls bordered by plasma membrane (Roberson et al., 2010). They are specialized dividing walls between cells found in almost all species of fungi. Dolipore septa, characteristic of basidiomycetes and first described by Moore and McAlear (1962), are characterized by a barrel-shaped swelling around their central pore, which is about 0.1–0.2 μm wide, typically capped at either end by specialized membranes, named “parenthosomes” (Figure 3d). TEM images have been widely used to verify the cell wall integrity such as in fungal mutants deleted in genes related to cell wall biosynthesis. This approach has been also used in combination with imaging tools such confocal fluorescence microscopy, X-Ray fluorescence and atomic force microscopy (Bakir et al., 2019), providing information at micro- and nano-scale. Particularly, atomic force microscopy provided quantification of ultrastructural cell wall architecture and near-field infrared spectroscopy allowed to spatially resolved chemical signatures, both at the nanoscale (Bakir et al., 2019).

## TEM Localization of Cell Wall Component

The use of *in situ* affinity techniques using specific probes, such as sugar-specific lectins or antibodies against fungal cell wall components conjugated with colloidal gold particles, has provided relevant information on the presence of several components (such as chitin, chitosan, and glucans) during the different growth stages (Balestrini and Bonfante, 2005, 2014; Figures 4a–c). Colloidal gold-conjugated lectins, such as concanavalin A (Con A) and wheat germ agglutinin, were used to label mannoproteins and chitin in yeast and filamentous fungi cell walls (Balestrini et al., 1996a). Chitin and β-1,3-glucans, the main carbohydrate components of fungal cell walls, have been localized in the cell walls of several ectomycorrhizal (Balestrini et al., 1996b, 2012; Martin et al., 1999) and endomycorrhizal fungi (Bonfante-Fasolo et al., 1990; Balestrini et al., 1994, 1996b, 2012; Lemoine et al., 1995) as well pathogens (Micali et al., 2011). Similarly, immunogold labeling with antibodies specific for β-1,3-glucans as well as β-1,6-glucans was used to localize the presence of glucans (Montijn et al., 1999) and chitosan (Maffi et al., 1998; Figure 4c) in the cell walls. TEM images allowed the localization of these polysaccharides in different the cell wall domains, showing that in yeast cell wall most of the α-1,3-glucan was along the cell membrane and appeared to enclose the cytoplasm (Sugawara et al., 2003). Immunogold experiments provided information about the presence of β-1,6-glucans in the yeast cell walls, showing they are lacking in the hyphae of ascomycete ectomycorrhizal *Tuber melanosporum* (Balestrini et al., 2012), independently from the growth stages (i.e., free-living or symbiotic stage). It is worth noting that various components from the cell walls can be dissected using specific glucanases before transmission electron microscopy (Hunsley and Burnett, 1970). However, Farkaš (2003) reported that enzyme dissection does not always allow straightforward interpretation of results since the cross-linking between the individual cell-wall components may result in the removal of the enzymically solubilized polymer as well as of other component(s) covalently linked to it.

## The Interface in Mycorrhizal Interactions

**Figure 5:**
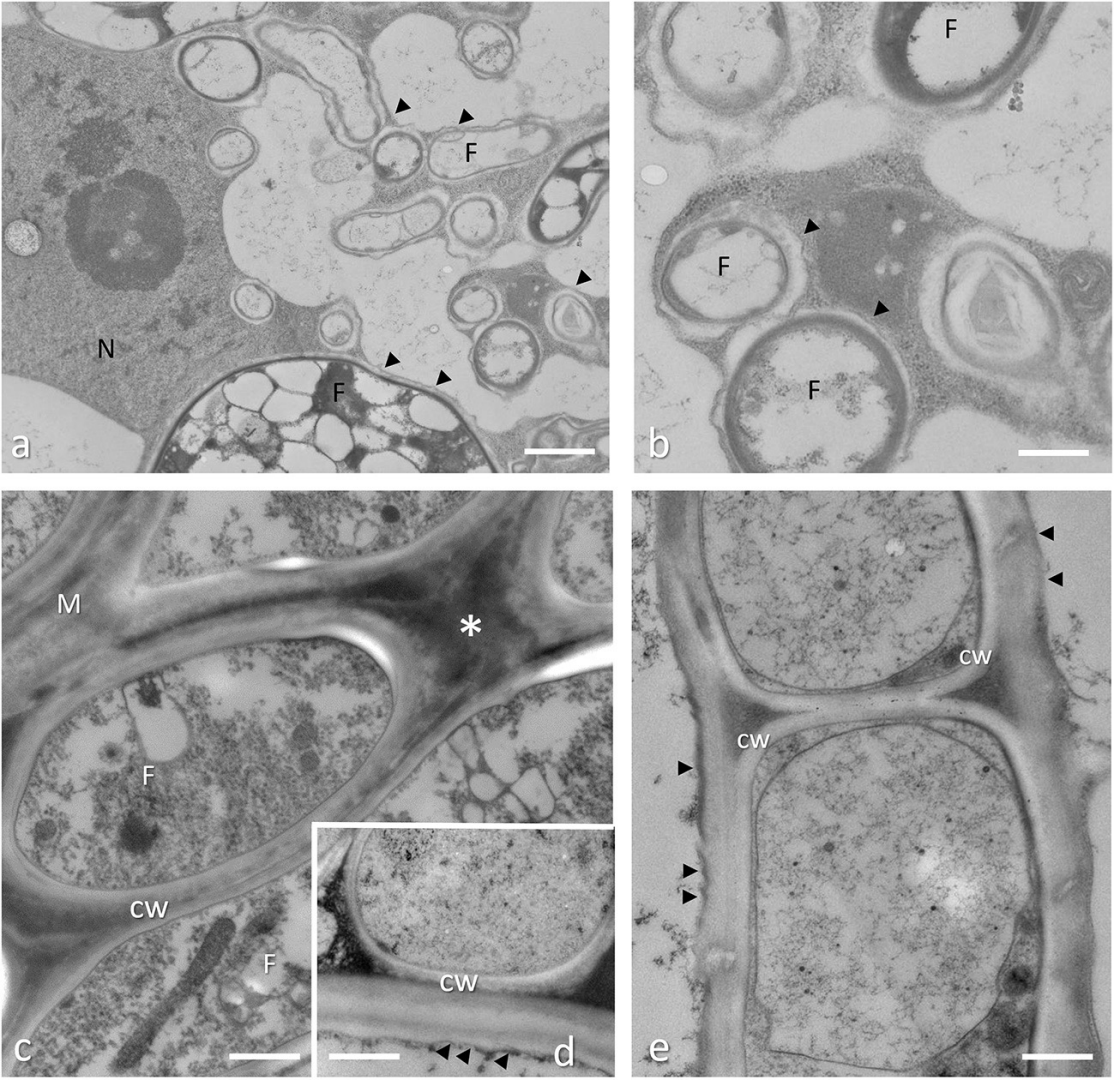
Arbuscular mycorrhizal root conventionally fixed **(a,b)** and *Tuber melanosporum* ectomycorrhizae **(c–e)**, cryofixed and freeze substituted. **(a)** During root colonization, the AM fungus reaches the inner cortical layers where is subjected to a peculiar branching process that leads to the highly branched structures, called arbuscules, which are considered the main site for nutrient exchanges. Bar = 2.0 μm. **(b)** At the electron microscope level, a new apoplastic space, based on membrane proliferation (arrowheads), is visible around the intracellular hyphae (F). Bar = 1.0 μm. **(c)** During *Quercus* root colonization, a fungal sheath around the host root (i.e., the mantle, m) is formed by ECM fungi. This structure is formed by packed hyphae with fungal cell wall (CW) that are in contact, which surrounds the host root cells. An electron-dense material is present among the mantle hyphae (asterisk). Bar = 0.3 μm. **(d)** Internal mantle, high magnification of a host-fungus contact. In ectomycorrhizal roots, the interface region between the partners is represented by the plant (arrowheads) and fungal cell walls (CW) that are in contact. Bar = 0.3 μm. **(e)** Hartig net (Hn) in a fully developed hazelnut ectomycorrhiza. Hyphae develop among plant cells, and fungal cell walls (CW) are in direct contact with the plant cell walls, showing a simple interface structure (arrows). Bar = 0.3 μm.

The interactions between roots and soil mycorrhizal fungi are an essential feature of the biology of most terrestrial plants (Smith and Read, 2008; Balestrini and Lumini, 2018). Mycorrhizal associations are divided into two main types basing on their ability to colonize the root cells: ecto- and endomycorrhizae that form two different plant-fungus interfaces (Balestrini and Bonfante, 2014). Detailed descriptions of the fungal structures formed inside or outside the plant roots are present in Peterson et al. (2004). Ultrastructural observations provided novel information on the development of endo- and ectomycorrhizal symbioses and the creation of the symbiotic interface (Balestrini and Bonfante, 2005, 2014). The presence of a new interface compartment is a typical feature of all endomycorrhizae, while the symbiotic interface in ECM appears much simpler, at least on a morphological level: the plant and fungal cell walls are always in direct contact with the ECM fungus that remains apoplastic (Balestrini and Kottke, 2016). In arbuscular mycorrhizal symbiosis, the fungus grows inter- and intracellularly all along the root, to spread fungal structures. When the fungus reaches the cortical layers, a peculiar branching process occurs, leading to the highly branched structures, called arbuscules, which are considered the main site for nutrient exchanges (Figures 5a,b). The AM fungus, as in other endosymbioses, is surrounded by a plant-derived membrane, which was called the periarbuscular membrane (arrowheads in Figures 5a,b), that leads to an interfacial zone. To reach the inner cortex, the AM fungus first needs to cross epidermal and outer cortical cell layers. Recent *in vivo* confocal imaging studies making use of fluorescent cellular markers have revealed that AM fungal penetration across epidermal cells is immediately preceded by the transient formation of a novel intracellular assembly called prepenetration apparatus (PPA) and comprising cytoskeletal and endoplasmic reticulum (ER) components (Genre et al., 2005). It includes a broad cytoplasmic bridge linking the site of fungal adhesion on the outer cell surface to the cell nucleus and defines the future intracellular path of hyphal penetration. Further *in vivo* observations showed that subsequent intracellular growth across the root outer cortex is PPA dependent. However, to gain further details on the PPA at ultrastructural level, carrot root segments containing inner cortical PPAs were first localized using a fluorescent ER probe and then prepared for TEM using a flat-embedding procedure (Genre et al., 2008). Ultrastructural observation of these PPA-containing cells revealed an intense membrane trafficking coupled with nuclear enlargement and remodeling, typical features of arbusculated cells, providing novel information at subcellular level with respect to those obtained by confocal microscopy alone (Genre et al., 2008). Electron microscope observations had already shown that the interfacial compartment in arbuscular mycorrhizal symbiosis contains cell-wall like material (Scannerini and Bonfante-Fasolo, 1983; Balestrini and Bonfante, 2005). Transmission electron microscopy observations, also in combination with colloidal gold methods, still represent an important tool to study fungal cell wall modification in native cell walls as well as during morphogenetic transitions. Thanks to TEM observations, it was possible to show that the cell walls of arbuscular mycorrhizal fungi undergo a conspicuous change in their organization during their life cycle: the spore wall is thick and layered with a highly fibrillar chitin, while the hyphal wall becomes progressively thinner during the intracellular phase, reaching a thin amorphous structure in the thinner arbuscular branches (Bonfante-Fasolo et al., 1990). In ectomycorrhizal interactions, during the invasion between living root cortical cells, the plant and the fungal walls are always in direct contact (Figures 5c–e). Using a Con A-gold conjugated complex, to localize high-mannose side chains of glycoproteins present in fungal cell wall, a labeling over the triangular electron-dense material present between the hyphae in the two ectomycorrhizal compartments (i.e., the mantle and the Hartig net) have been found, suggesting that this cementing material may contain a fungal wall component (Balestrini et al., 1996a). Fungal cell wall proteins (e.g., SRAPs and hydrophobins) have been also localized in the cell walls at the symbiotic interface in ectomycorrhizal interactions (Laurent et al., 1999; Tagu et al., 2001), supporting the biochemical data that have shown a differential expression of fungal cell wall proteins during ectomycorrhizal interactions (Laurent et al., 1999; Martin et al., 1999; Tagu et al., 2001). Additionally, a secreted phospholipase A_2_ (TbSP1), which is upregulated by nutrient starvation as well as during the symbiotic phase, has been localized in the inner cell-wall layer of the ectomycorrhizal fungus *Tuber borchii* Vittad. (Soragni et al., 2001; Miozzi et al., 2005), and a fungal expansin- like protein, whose expression is specific to symbiotic tissues in *Laccaria bicolor*-*Populus* ectomycorrhizae, has been localized within fungal cell walls, thus suggesting a role in fungal cell wall remodeling during symbiosis establishment (Veneault-Fourrey et al., 2014).

## Infection Structures of Plant Pathogenic Fungi

Plant pathogenic fungi, on the basis of their trophic relationship with the host, are divided into biotrophic, hemibiotrophic, and necrotrophic (Heath, 1983). The latter do not have specific infection structures as their hyphae directly secrete enzymes and toxins to kill host tissues, therefore adsorbing nutrients from dead cells. Hemibiotrophic fungi, may penetrate their host plants directly through a fine hypha produced by the spore or mycelium. This hypha, once inside, can directly absorb nutrients from the host, initially without damaging it excessively and keeping cells and tissues alive, like typical biotrophic fungi. Afterwards, when the mycelium has developed adequately, it generates hyphae capable of secreting large quantities of toxins and enzymes, as the necrotrophs do. All these infection steps have been reconstructed and elucidated mainly by ultrastructural studies on *Colletotrichum lindemuthianum* (Sacc. & Magn.) Bri. & Cav., carried out by O'Connell and co-authors in 1980s, also with the pioneering use of high pressure freezing followed by freeze substitution and immunolabelling with antibodies or lectins to recognize specific fungal macromolecules (O'Connell, 1987; O'Connell and Bailey, 1991; Pain et al., 1994).

True biotrophic fungi, which maintain their lifestyle during the whole infection process, develop instead specific structures, i.e., haustoria, for parasitism with the aim of keeping host cells alive, as far as possible. At this regard, the study by TEM of ultrathin sections of the interface fungus-host plant has been fundamental in clarifying how this biotrophism can be established, particularly regarding the up-take of nutrients by the haustoria without incurring in lethal damages of plant cells. The first TEM images of haustoria dated back to early 1960s and were about the Basidiomycota *U. caladii* and *Puccinia graminis* Pers. (Moore and McAlear, 1961; Ehrlich and Ehrlich, 1963). Since then, the progressive improvement of fixation and embedding techniques, coupled with cytochemical and enzymatic detection of macromolecules, allowed a better comprehension of the structure and function of this interface. A great contribution in this sense was made among all by the studies of Chong and Harder (1980), Chong et al. (1981, 1986), Heath (1976, 1983, 1987), and Mendgen (1975, 1979). Another decisive step was the introduction in 1980s of high pressure freezing and cryosubstitution that often resulted in dramatic improvement of ultrastructural details of the haustoria and surrounding host plasma membrane, i.e., showing that the latter is linear and not wavy as an artifact of chemical fixation (Hippe, 1985; Knauf and Mendgen, 1988; Mims et al., 2002). From all the above studies we can now describe in detail how the establishment of the host-pathogen interface occurs in the case of Ascomycota and Basidiomycota, the most economic important biotrophic fungi.

**Figure 6:**
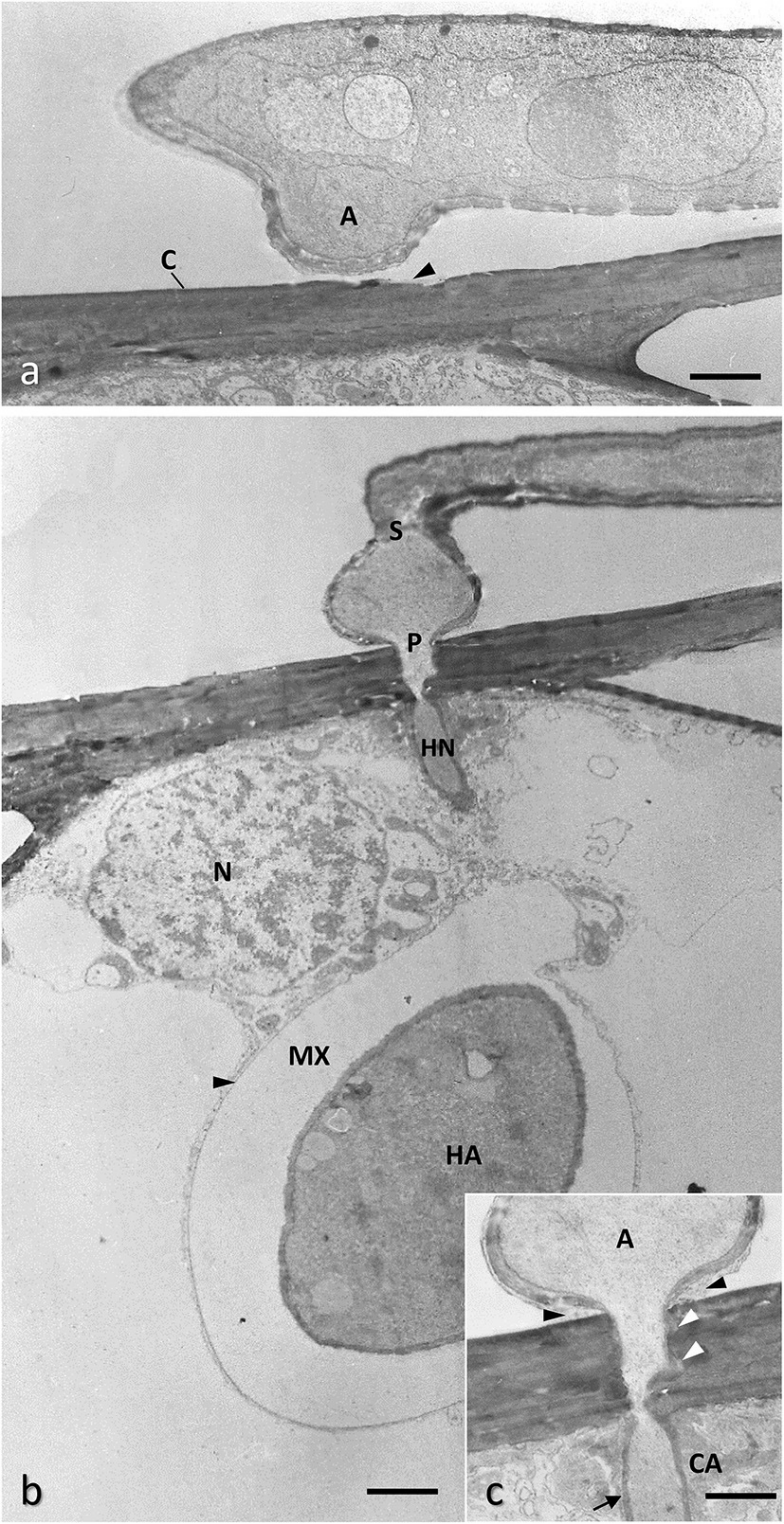
Conventionally chemical fixed samples of barley epidermal cell infected with *Blumeria graminis* f.sp. *hordei* showing the early phase of the infection process. **(a)** A hypha growing on the leaf surface is generating an appressorium (A) encountering the plant cuticle (C) which appears already eroded (arrowhead) by fungal hydrolyzing enzymes. Bar, 1.2 μm. **(b)** The appressorium, once separated by the hyphae by a septum (S) generates a penetration peg perforating the host cell wall and developing an infection hyphae (haustorial neck, HN) inside the host cell; this hyphae force the plasma membrane (black arrowheads) to invaginate without breaking it, then expands to form the haustorium (H) in which the content of the appressorium is transferred through the haustorial neck, part of which is not visible here because out of the section plane; the forming haustorium is separated by the host membrane by an extra-haustorial matrix (MX) rich in polysaccharides; the nucleus (N) of the host cell move close to the haustorium to facilitate transcriptional reprogram of the infected cell; courtesy of D. Maffi. Bar, 1.6 μm. **(c)** Enlargement of the penetration point showing adhesive material fixing the appressorium to the cuticle (black arrowheads); the host cell wall around the penetration peg appears loosen (white arrowheads) and a callose collar (CA) is formed around the haustorial neck, whose cell wall is thickened (harrow). Bar, 1.0 μm.

**Figure 7:**
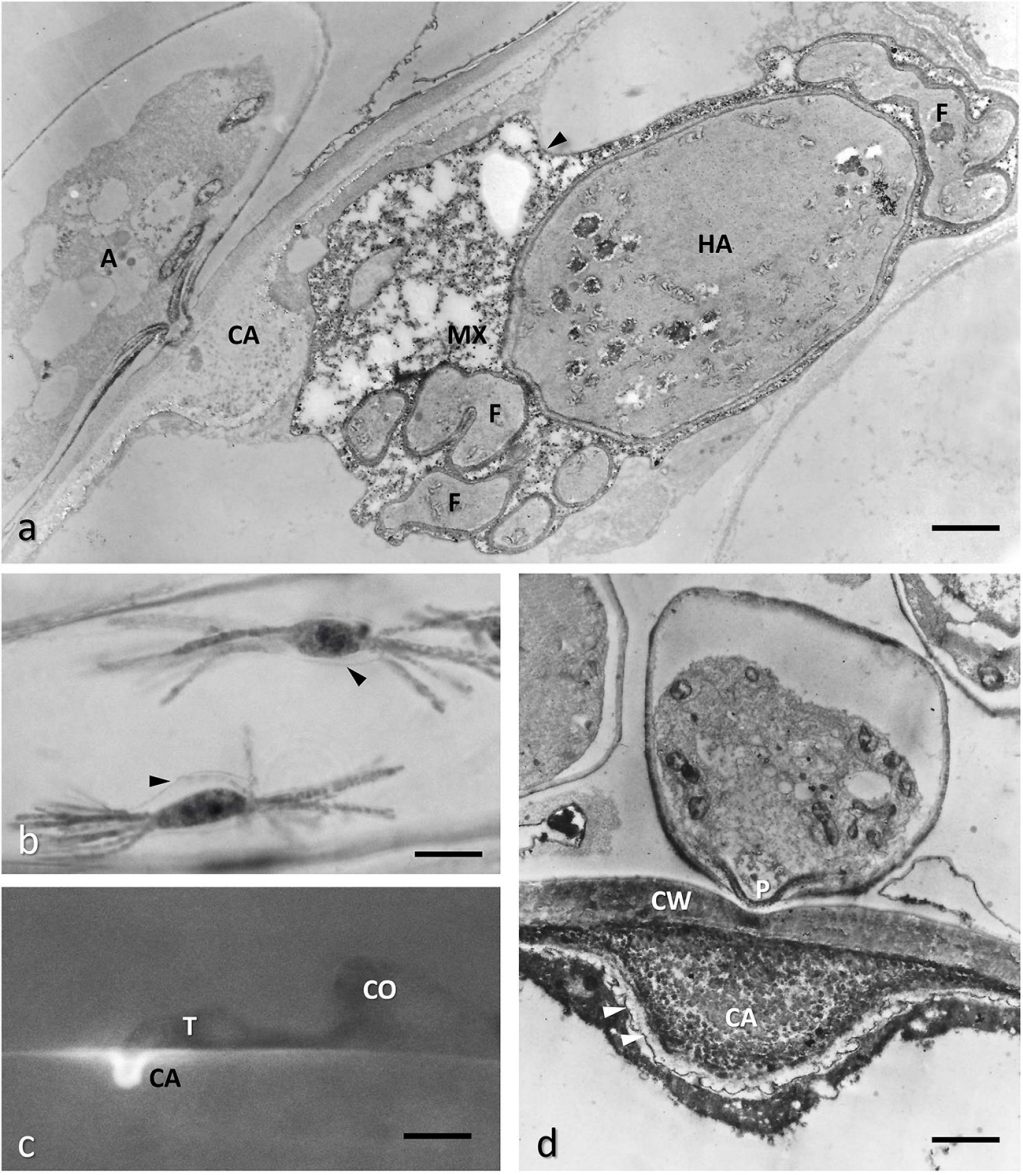
Barley epidermal cell infected with *Blumeria graminis* f.sp. *hordei* showing different phases of the infection process. **(a)** TEM image of a conventionally fixed haustorium (HA), fully developed with numerous finger-like protrusions (F); the host membrane surrounding HA, also called extra-haustorial membrane, appears thickened (arrowheads); the extra-haustorial matrix (MX) contains granular and amorphous materials; the haustorial neck is out of the section plane, however a large callose collar (CA) is visible at the penetration site; the protoplasm of the appressorium (A) is moving to the HA. Bar, 1.6 μm. **(b)** Interference-contrast light microscopy of two fully expanded haustoria in an epidermal cell showing finger-like protrusions and the thickened host plasma membrane surrounding them (arrowheads). Bar, 20 μm. **(c)** Fluorescence microscopy of a penetration attempt stopped by the apposition of a callose papilla (CA, stained with aniline blue) by a resistant barley cultivar; the attempt is made by the germ tube (T) of the fungus from a germinated conidium (CO). Bar, 5 μm. **(d)** TEM image of the callose papilla: note that it is formed as soon as the penetration peg (P) starts to perforate the host cell wall (CW); the papilla is filled also with electron-dense material, possible polyphenols, which are also deposited along the host plasma membrane (arrowheads); courtesy of D. Maffi. Bar, 0.14 μm.

Pathogenic Ascomycota are mainly represented by powdery mildew fungi that are worldwide diffused and caused severe damages to many crops. Among them, *Blumeria graminis* (DC.) Speer, by its many specialized races infect almost all cereals, *Podosphaera fuliginea* (Schltdl.) U. Braun & S. Takam (formerly *Sphaerotheca fuliginea* Schlechtend.:Fr., Pollaci) affects cucurbits and *Erysiphe necator* Schwein (formerly *Uncinula necator* Schwein., Burrill) causes one of the main fungal diseases in the vineyards (Agrios, 2005). These epiphytic parasites penetrate only the epidermal cells, thus constituting a convenient experimental model for studying the plant cell-haustorium interface. Powdery mildew hyphae break into the host through a penetration peg produced on epidermal cells by an appressorium (Emmett and Parbery, 1975) (Figures 6a,b) which is formed at the point of contact of the germ tube of a spore or a proliferating mycelium with the plant cuticle. The role of the appressorium, usually bulbous or cylindrical with a flat surface (Figures 6a,b), is to hold the penetration peg firmly during penetration. This is achieved by secreting adhesive glycoproteins and polysaccharides on its surface in contact with the host (Agrios, 2005). The penetration peg pierces the host cuticle and the cell wall by a combination of mechanical force and enzymatic softening of the cell wall substances structures (Price-Jones et al., 1999) (Figure 6c). The hypha narrows as it passes through the host cell wall and then expands between the wall and the plasma membrane without breaking the latter to avoid cell death (Figures 6b, 7a). This expansion leads to the formation of the haustorium, a feeding organ that often has a simple globose form but in some fungal species, i.e., *B. graminis*, can also form finger-like protrusions and branching to augment the adsorbing surface (Aist and Bushnell, 1981) (Figures 7a,b). The plasma membrane surrounding the haustorium is also called extrahaustorial membrane (EHM) and becomes highly modified in the invaginated zone. Where the haustorial neck breaches the host cell wall, a collar of callose is secreted all around (Figures 6c, 7a–c) and in resistant cultivars the high amount of callose and other materials, i.e., polyphenols, may form a so-called papilla that completely stop haustorium development or, at least, encase it (Aist and Israel, 1986) (Figures 7c,d). The ATPase activity of EHM is inhibited by the fungus to facilitate diffusion of nutrients from host protoplasm toward the fungus itself. Furthermore, at the level of the haustorial neck the EHM appear very thick and electron-dense, possibly to prevent that adsorbed solute diffuse through the haustorial cell wall at neck level (Figure 6c) (Lucas, 1998). The region between EHM and fungal cell wall is called extrahaustorial matrix and at TEM appears as formed by amorphous material, usually scarcely electron-dense in early phase of haustorium development and later showing aggregations of granular or amorphous material, possibly secreted by the host and forming a sort of exchanging/filtering layer between the two organisms (Aist and Bushnell, 1981) (Figure 7a).

**Figure 8:**
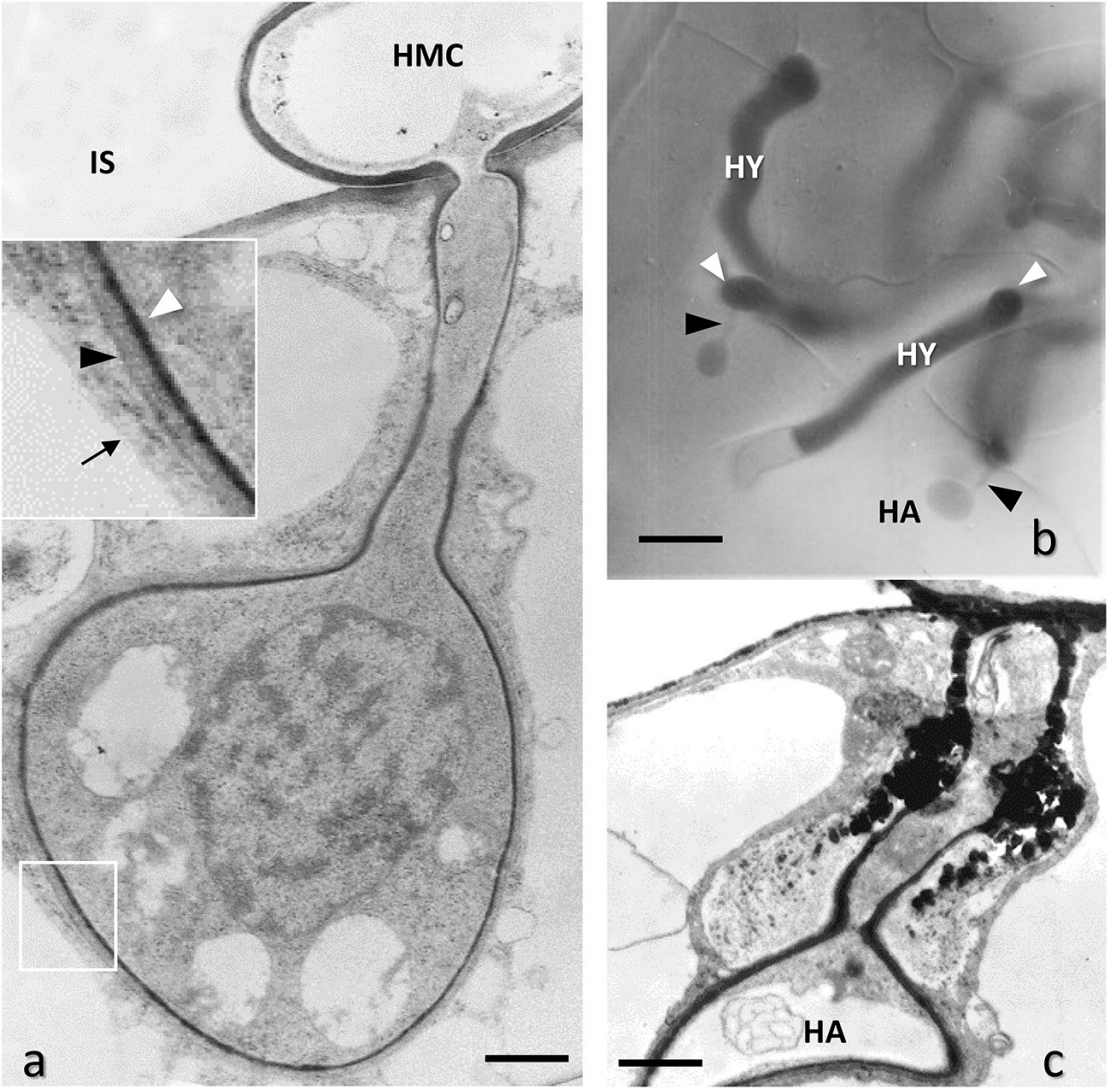
Leaf mesophyll cells of *Phaseolus vulgaris* infected with bean rust by *Uromyces appendiculatus*, Basidiomycota. **(a)** TEM image of a globose haustorium (HA) generated by a haustorial mother cell (HMC) in the intercellular space (IS); the framed area is enlarged in the inset, showing a thin layer of an extra-haustorial matrix between a fungal cell wall (white arrowheads) and host plasma membrane (black arrowhead), the latter appearing thickened, while the close tonoplast membrane (arrow) is normal. Bar, 1.0 μm. **(b)** Interference-contrast light microscopy of the fungal hyphae growing in the mesophyll intercellular space and generating haustoria (HA) connected to the haustorial mother cell by a thin neck (black arrowheads). Bar, 10 μm. **(c)** TEM image of a collapsing haustorium (HA) due to the secretion of toxic polyphenols (PH) in the extra-haustorial matrix around the neck by plant resistance mechanisms. Bar, 1.0 μm.

Contrary to powdery mildew, which penetrates the epidermal cells forming haustoria inside them, some Basidiomycota, i.e., urediniospores of the rust fungi *Puccinia graminis* f. sp. *tritici* Erikss. & Henning and *Uromyces appendiculatus* (Pers.) Link, and zoospores of some fungal-like Oomycotes, i.e., *Plasmopara viticola* (Berk. & M. A. Curtis) Berl. & De Toni, penetrate through stomata and form haustoria in mesophyll cells (Chong and Harder, 1980; Burruano, 2000; Maffi et al., 2011; Solanki et al., 2019) (Figures 8a,b). These fungi have the double advantage of absorbing nutrients both through the haustorium and the hyphae that colonize the intercellular spaces. In this case the formation of the haustorium occurs from haustorium mother cells (HMC) which develops from hyphae proliferating in the intercellular spaces of the leaf (Knauf et al., 1989) (Figures 8a,b). HCM function is the same as for powdery mildew appressorium, thus holding the penetration peg firmly during penetration. The haustorium is bulbous, often almost spherical and its neck quite long (Figures 8a,b), being mesophyll cells generally larger than epidermal cells. In any case its structure is like bulbous powdery mildew haustoria, however with a thinner extrahaustorial matrix isolating fungal cell wall from host plasma membrane (Chong and Harder, 1980) (EHM) (Figure 8a, inset). The latter appears thicker than in the other parts of the cell due to the alteration induced by the fungus, as described above. The outstanding preservation of ultrastructural details afforded by high-pressure freezing followed by freeze substitution of *Puccinia hemerocallidis* Thüm haustoria, coupled with the use of gold-conjugated wheat germ agglutinin for labeling of chitin, revealed that the electron-dense material present in the extrahaustorial matrix were not part of the haustorial wall, but likely of the host. At this regard, the EHM showed points of continuity with electron-dense tubular elements present in the host cytoplasm nearby the haustorium, as well as with flattened cisternae, possibly as a consequence of the host endomembrane rearrangement in the form of smooth endoplasmic reticulum (Mims et al., 2002). As for powdery mildew, plant defense mechanisms may hamper haustoria formation, even in the late phase of haustorium development (Maffi et al., 2011), i.e., secreting callose and toxic polyphenols in the extrahaustorial matrix, causing haustorium necrotization (Figure 8c). In this regard, it should be pointed out that the ultrastructural studies at TEM, possibly conjugated with other microscopical techniques and biochemical studies, still remain fundamental both in the study of the defense mechanisms of plants from fungal diseases and in the study of the mechanisms of action of new fungicide (Sun et al., 2020; Lorrai et al., 2021; Wang et al., 2021).

## Conclusions

In conclusion, ultrastructure analyses have extensively contributed to highlight fungal features correlated to cell organization and growth. The potential of transmission electron microscopy to study the fungal ultrastructure has been accompanied by development of more advanced techniques, such as freeze-fracturing, microautoradiography and the application of affinity techniques using lectins or antibodies in conjugation with colloidal gold particles against defined cell-wall epitopes. More recently, TEM observations might advantage or may represent a support for next-wave single cell analyses that provide highly resolved structural and compositional features of biological specimens at micro- and nano-resolution. Thanks to detailed works at TEM and the use of affinity techniques, information on plant-fungal interactions has been also provided. If in the past the spread of the molecular biology approaches allowed to explain what was observed, nowadays, after the huge of information obtained from diverse *omics* techniques on fungi belonging to diverse groups, ultrastructural studies offer the possibility to support the molecular data. Although in the last years, mainly thanks to the data obtained in the frame of fungal genome projects, several novel information has been obtained. The full comprehension of the mechanisms at the basis of the fungal growth and the interaction with other organisms, is in fact closely related to a detailed knowledge of the structural features.

## Author Contributions

FF and RB conceived and wrote the manuscript. AF performed high pressure freezing experiments on truffle mycelium and ectomycorrhizae. All authors contributed to the article and approved the submitted version.

## Conflict of Interest

The authors declare that the research was conducted in the absence of any commercial or financial relationships that could be construed as a potential conflict of interest.

## Publisher's Note

All claims expressed in this article are solely those of the authors and do not necessarily represent those of their affiliated organizations, or those of the publisher, the editors and the reviewers. Any product that may be evaluated in this article, or claim that may be made by its manufacturer, is not guaranteed or endorsed by the publisher.
