# Supplementary material for: Contributions of Ultrastructural Studies to the Knowledge of Filamentous Fungi Biology and Fungi-Plant Interactions
Source: Front Fungal Biol. 2022 Jan 24;2:805739. doi: 10.3389/ffunb.2021.805739 (PMC10512230; doi:10.3389/ffunb.2021.805739)
Supplement: Supplementary file 1 [file Data_Sheet_1.docx]

**CONTRIBUTIONS OF ULTRASTRUCTURAL STUDIES TO THE KNOWLEDGE OF FILAMENTOUS FUNGI BIOLOGY AND FUNGI-PLANT INTERACTIONS**

Franco Faoro*^1^, Antonella Faccio^2^ & Raffaella Balestrini*^2^

^1^Dipartimento di Scienze Agrarie e Ambientali, Università di Milano, Via Celoria 2, 20133 Milano

^2^Consiglio Nazionale delle Ricerche, Istituto per la Protezione Sostenibile delle Piante, Strada delle Cacce 73, 10135 Torino

**Appendix**

The methods described below are the result of many decades of work by the authors in studying the ultrastructure of fungi and their infection process in plants. They derive mainly from the original fixation and embedding methods developed by the pioneering work of Glauert (1965, 1975, 1991) and from the immunolabeling techniques introduced by Bendayan (1981, 1984), Newman and Hobot (1987, 1999). A comprehensive collection of these techniques can be found in Hayat (1989) and in Newman and Hobot (2001). As regards high pressure freezing and freeze-substitution protocols to study fungal hyphae ultrastructure as well as the fungal structures involved in symbiotic and pathogenic plant interactions have been reported by Roberson (1993, 2010) and Mendgen (2000).

1. **Conventional fixation and embedding methods for studying fungal ultrastructure**

For fungi growing on substrates (i.e. solid media, host tissues) small sample fragments (1-2 m^3^) must be excided in the primary fixative. For fungi growing in liquid media or spores and other fungal propagules primary fixation can be performed in small centrifuge tubes and samples spun down afterwards, then embedded in 2% aqueous agar at 40°C for further processing.

- Primary fixation (2 hr or more at room temp.) in a mixture of paraformaldehyde (1-4%) and glutaraldehyde (2-3%) in cacodylate or phosphate buffer 0.1 M, pH 6.9-7.4
- Wash (30-60 min) in the same buffer
- Post-fixation (2hr or more) in 1% osmium tetroxide in the same buffer or distilled water
- Wash in the same buffer (10-15 min)
- Dehydration in an ethanol series (i.e. 25%-50%-75%-90%), 15 min each step, then in absolute ethanol for 1 hr
- Infiltration with an absolute ethanol/Spurr resin (Spurr, 1969) mixture 3:1, 1:1 and 1:3 for 1hr each step, then in pure resin for at least 12 hr
- Resin polymerization in suitable moulds at 60 °C for 24 hr.
- Ultrathin sectioning (60-100 nm) and staining with aqueous uranyl acetate (1-2%) and lead citrate (Reynolds, 1963)

1. **Immunolocalization of macromolecules with colloidal gold at TEM**

Fungal samples are taken and processed as described in the previous section with the following modifications: fixation in paraformaldehyde 4% and glutaraldehyde 0.5-1.0% for up 2 hr at 4°C, post-fixation to be omitted and Spurr/Araldite resin replaced by London Resin White (Newman, 1987), polymerised in gelatine capsules sealed without trapped air (Moore et al. 1991).

Ultrathin sections, collected with nickel grids (100-200 mesh) coated with collodion film are usually labelled with a two steps method with a primary antibody to recognize the target and a commercial gold-conjugated secondary antibody to recognize the primary antibody, as below.

- Incubate grids for 15 min at 37°C in a humid chamber on a drop (30-40 µL) of PBS (phosphate buffered saline, 0.01M, pH 7.4) added of 0,05 % BSA (bovine serum albumin);
- Move grids on a drop of a primary antibody (i.e. raised in rabbit) diluted 1:10-1:1000 (depending on the serum titre) with PBS and incubated for 1-3 hr at 37°C or overnight at 4°C;
- Wash thoroughly with PBS and move grids on a 20 drop of gold-conjugated secondary antibody (i.e. goat-antirabbit) diluted in PBS 1:20 (or as suggested by the manufacturer)
- Incubate for 1hr at 37°C then wash grids thoroughly with PBS and finally with distilled water, letting them drying before staining with uranyl acetate and lead citrate

N.B. Do not let section dry at any step of immunolocalization, only drain out the excess of solution; the choice of gold particle size depends on the required magnification of specimens, i.e. 10-20 nm are suitable for 10-20 K magnifications.

**3. High pressure freezing and freeze-substitution to improve preservation of fungal ultrastructure**

Fungi growing on substrates (i.e., solid media, host tissues) or in liquid media or spores and other fungal propagules are transferred in the specific aluminium cavity of the apparatus (e.g., HPM 010 - Balzer Union or HPM100 Bal-Tec products), filled with the cryoprotectant 1-hexadecene, and rapidly freezed in liquid N_2_ under a pressure of 2100 bar that depresses the melting point of water retarding the growth rate of the dangerous ice crystals. Frozen samples are stored in liquid nitrogen until further processing.

The frozen samples must be subjected to freeze-substitution to replace the ice inside with another solvent and a fixative, whilst keeping specimens at low temperature with the follow steps (as an example):

- Incubate at -85° C for 48 to 72 hr in 1.0% glutaraldehyde + 1.0% tannic acid, in dry acetone
- Rinse in acetone at -85° C three times
- Transfer to cold (-85° C) 1.0 % OsO_4_ in acetone and incubate for 1 hr, then raise the temperature at -20° C for 2 hr and at 4° C for more 2 hr
- Transfer specimens at room temperature for 1 hr then rinse in acetone three times
- Infiltrated in a mixture of epoxy resin (i.e., Epon/Araldite, Spurr) and acetone, very slowly starting with 10% of resin for 1 hr and raising the resin amount by 10% every hour up to 80%, then leave overnight
- Change the mixture with a fresh 80% resin in 20% acetone for 1h then raise the resin to 90% for 1h before transferring specimens in pure resin for 3 hr with two intermediate changes
- Polymerise the resin in moulds at 60°C for 24 hr.

In case the specimens should be immunolabelled as described in section 2, glutaraldehyde concentration must be reduced to 0.2%, tannic acid replaced by 0.1% uranyl acetate and OsO_4_ omitted. Furthermore, the epoxy resin must be replaced by an acrylic resin such as London Resin White with the same infiltration and embedding steps as above.

**References only present in the Appendix**

Bendayan, M. (1981). Ultrastructural localization of nucleic acids by the use of enzyme gold complexes. Histochem. Cytochem. 29, 531-541.

Glauert, A.M. (1965). The fixation and embedding of biological specimens. In: Kay D (ed.) Techniques for Electron Microscopy, 2nd. edition. Blackwell Scientific Publications, Oxford, pp. 166-212

Glauert, A.M. (1975). Fixation, Dehydration, and Embedding of Biological Specimens. Practical Methods in Electron Microscopy, Vol.3, Part I. Elsevier/North Holland, Amsterdam

Glauert, A.M. (1991). Epoxy resins: an update on their selection and use. Microscopy and Analysis 25, 15-20.

Hayat, M.A. (1989). Colloidal Gold: Principles, Methods, and Applications, Vol.2. Academic Press, San Diego.

Mendgen, K. (2000). High-Pressure Freezing to Study Structure and Function of the Host Parasite Interface. Microscopy and Microanalysis, 6(S2), 682-683.

Moore, P.J., Swords, M.M., Lynch, M.A., Staehelin, L.A. (1991). Spatial organization of the assembly pathways of glycoproteins and complex polysaccharides in the Golgi apparatus of plants. J. Cell Biol. 112, 589-602.

Newman, G.R. (1987). Use and abuse of LR White. Histochemistry, 19, 118-120.

Newman, G.R., Hobot, A. (1987). Modern acrylics for post-embedding immunostaining techniques. Histochem. Cytochem., 35, 971-981.

Newman, G.R., Hobot, J.A., (1999). Resins for combined light and electron microscopy: a half century of development. Histochemistry, 3, 495-505.

Newman, G.R., Hobot, J.A., (2001). Resin microscopy and on-section Immuno-cytochemistry. Springer Lab Manuals, second edition, Springer, Berlin, 273 pp.

Reynolds, E. S. (1963). The use of lead citrate at high pH as an electron opaque stain

in the electron microscope. J. Cell Biol. 17, 208-212.

Roberson, R.W. (1993) Cryofixation and freeze substitution of teliospores of *Gymnosporangium clavipes*: an ultrastructural investigation. Mycol. Res. 97, 195-204.

Spurr, A.R. (1969). A low-viscosity resin embedding medium for electron microscopy. J. Ultrastruct. Res., 26, 31-43.
